# Nutritional Improvements of Sourdough Breads Made with Freeze-Dried Functional Adjuncts Based on Probiotic *Lactiplantibacillus plantarum* subsp. *plantarum* and Pomegranate Juice

**DOI:** 10.3390/antiox12051113

**Published:** 2023-05-18

**Authors:** Stavros Plessas, Ioanna Mantzourani, Athanasios Alexopoulos, Maria Alexandri, Nikolaos Kopsahelis, Vasiliki Adamopoulou, Argyro Bekatorou

**Affiliations:** 1 Laboratory of Food Processing, Department of Agriculture Development, Democritus University of Thrace, 68200 Orestiada, Greece; imantzou@agro.duth.gr; 2 Laboratory of Food Biotechnology, Microbiology and Hygiene, Department of Agriculture Development, Democritus University of Thrace, 68200 Orestiada, Greece; alexopo@agro.duth.gr; 3 Department of Food Science and Technology, Ionian University, 28100 Argostoli, Greece; malexandri@ionio.gr (M.A.); kopsahelis@ionio.gr (N.K.); 4 Department of Chemistry, University of Patras, 26504 Patras, Greece; adamopoul_v@upatras.gr

**Keywords:** *Lactiplantibacillus plantarum* subsp. *plantarum* ATCC 14917, sourdough bread, pomegranate juice, antioxidant capacity, phenolic content, phytic acid

## Abstract

New types of sourdough breads are proposed, made with freeze-dried sourdough adjuncts based on: (i) *Lactiplantibacillus plantarum* subsp. *plantarum* ATCC 14917, a potential probiotic (LP) alone or (ii) with the addition of unfermented pomegranate juice (LPPO) and (iii) pomegranate juice fermented by the same strain (POLP). Physicochemical, microbiological, and nutritional characteristics (in vitro antioxidant capacity, AC, total phenolics, TPC, and phytate content) of the breads were evaluated and compared with commercial sourdough bread. All adjuncts performed well; the best results being those obtained by POLP. Specifically, the highest acidity (9.95 mL of 0.1 M NaOH) and organic acid content (3.02 and 0.95 g/kg, lactic and acetic acid, respectively) as well as better resistance to mold and rope spoilage (12 and 13 days, respectively) were observed for POLP3 bread (sourdough with 6% POLP). Significant nutritional improvements were observed by all adjuncts, in terms of TPC, AC, and phytate reduction (103 mg gallic acid/100 g, 232 mg Trolox/100 g, and 90.2%, respectively, for POLP3). In all cases, the higher the amount of adjunct, the better the results. Finally, the good sensory properties of the products indicate the suitability of the proposed adjuncts for sourdough breadmaking, while their application in freeze-dried, powdered form can facilitate commercial application.

## 1. Introduction

Bread and bakery products represent the most popular and essential foods of the human diet worldwide. The current commercial and research trends related to breadmaking focus on the extension of the shelf life by inhibiting microbial spoilage and physicochemical quality downgrading (staling), as well as on the improvement of the nutritional value of bread. Although many biotechnological approaches have been proposed, the application of various sourdough techniques seems to be effective for achieving the above goals [1,2,3,4]. The available extensive literature indicates that sourdough can improve the overall quality of bread while increasing its nutritional value; however, the discovery and proper use of defined sourdough starter cultures is essential.

Innovations in food production also focus on the development of functional foods, including bread and bakery products [5]. The example of gluten-free bread is among the most popular innovations [6]. Others focus on the reduction in the glycemic index and calories, the increase in bioactive phytochemicals and antioxidant capacity (AC), and the decrease in antinutrients such as phytate. Likewise, the development of new functional bakery products as vehicles of bioactive ingredients for the delivery of health benefits to consumers has lately gained attention [2,5,7].

The dietary antioxidants include phenols, ascorbates, tocopherols and carotenoids. Phenolic phytochemicals in particular are known for both their AC and antimicrobial activity, while they may also exhibit anti-inflammatory, anti-allergic, and anti-proliferative properties. Likewise, a diet that includes high levels of phenolic phytochemicals has been correlated with important health benefits [8]. Nevertheless, food matrices are complex mixtures of macro- and micro-components forming structures that are able to modulate or even inhibit the activity of phenolic compounds [9]. Cereal grains contain phenolic compounds, which explains their enhanced AC [8,10]. However, during bread production, a multitude of chemical reactions takes place involving its main components and bioactive phytochemicals, such as protein denaturation, starch gelatinization, the thermal degradation of sugars and amino acids (Strecker and Maillard reactions), etc. At the same time, new compounds appear, altering the bioactive and sensory properties of bread [8,11].

Since bread is a universal staple food, its supplementation with phenolic compounds has been extensively studied. Rye and wheat are the basic grains for bread production, and their fortification with other grains or/and bioactive substances has been studied. Typical examples are the enrichment of wheat bread with oat flour [12] and the production of barley bread [13], resulting in products with elevated antiradical activity and AC. Non-cereal seeds, vegetables, and fruits have also been used as a source of antioxidants for bread enrichment [14]. The supplementation of breads with fruits has shown a significant potential to increase their AC while improving their sensory properties. The available literature includes the addition of grape seed extract [15], blackcurrant polyphenols combined with pectin [16], cherry fruit powder [17], and apple, plum, carrot and cabbage powder [18].

Bread enrichment with pomegranate has also been evaluated with encouraging results. For example, a study demonstrated that fiber-enriched bread with good sensory and technological qualities can be produced by the addition of 5% pomegranate seed flour in wheat flour [19]. Additionally, a study on the nutritional, rheological, and sensory quality of bread made with white flour enriched with powdered cinnamon and pomegranate peel showed a slight decrease in moisture and protein contents, while the ash, fiber and radical scavenging activity were increased [20]. The study of the effect of the fermentation time and baking temperature on the AC and phenolic content of bread enriched with grape and pomegranate seed also showed that the baking process significantly influences the bioactive components and AC [21]. Finally, the effect of the consumption of wheat bread fortified with pomegranate peel powder was assessed on patients with type 2 diabetes, indicating the potential of such an intervention on glycemic indices, the antioxidant status, inflammation and mood [22].

Recently, our research group successfully fermented pomegranate juice by *Lactiplantibacillus plantarum* subsp. *plantarum* ATCC 14917 to produce a good-quality, low-alcohol fruit beverage with high nutritional value in terms of the AC and total phenolic content (TPC). These features, as well as the viability of the strain, were retained during storage [23]. *L. plantarum* strains are considered to be versatile and adaptable lactic acid bacteria (LAB), with claimed probiotic properties [3]. Several studies have demonstrated their various effects on bread such as the extension of the shelf life, the production of antimicrobial metabolites (organic acids and antifungal compounds), the production of peptidases that can degrade gluten, the production of peptides and amino acids that improve the nutritional and sensory quality of bread, and the expression of other enzymes that can affect the breadmaking process (aspartate ammonia-lyase, phenolic acid esterase, decarboxylases, reductases, glycosyl hydrolases, etc.) [3]. Based on these findings, it was considered interesting to combine the effects of a probiotic *L. plantarum* strain and pomegranate juice to produce sourdough bread.

Therefore, the aim of the present study was to examine, for the first time, (1) the use of the potentially probiotic stain *L. plantarum* subsp. *plantarum* ATCC 14917 as a starter in sourdough breadmaking, and the use of novel, nutritious supplements that consist of (2) pomegranate juice fermented by the same strain or (3) the freeze-dried plus unfermented freeze-dried pomegranate juice. Both the starter and the supplements were used in freeze-dried, powdered form to evaluate their use as dried commercial adjuncts in breadmaking. The effect of the adjuncts on various physicochemical (pH, acidity, loaf volume), nutritional (TPC, AC, and phytate contents), sensory, and microbiological properties (rope and mold spoilage) of the produced sourdough breads was examined.

## 2. Materials and Methods

### 2.1. Microorganism and Media

The probiotic strain *L. plantarum* subsp. *plantarum* ATCC 14917 (referred to as *L. plantarum* from this point on) was grown under anaerobic conditions at 37 °C for 48 h in sterile de Man, Rogosa and Sharpe (MRS) broth (Fluka, Buchs, Switzerland), and it was harvested by centrifugation (Sigma 3K12, Βioblock Scientific, Sigma Larborzentrifugen GmbH, Osterode, Germany) at 5000 rpm for 10 min [23].

The preparation of pomegranate juice was as described previously [23]. In brief, the seeds of fresh pomegranates (*Punica granatum* L.) were processed into juice by blending in a household blender, the sugar concentration was adjusted to 80 g/L by the addition of sterilized, deionized water, and the juice was pasteurized (5 min/80 °C) and cooled to room temperature (18–20 °C) before use.

Commercial white flour was used for breadmaking (Hellenic Biscuit CO S.A., Acharnes, Greece). It contained (% *w*/*w*): 11.0% protein, 72.0% carbohydrates, 1.5% fat, 2.2% fiber and a 12.0% moisture content.

### 2.2. Fermentation of Pomegranate Juice

The fermentation of pomegranate juice was performed at 37 °C for 48 h using 1 g of harvested (wet weight) *L. plantarum* cell mass per 100 mL of pomegranate juice, as previously described [23].

### 2.3. Freeze-Drying

Freeze-drying of the *L. plantarum* cell mass, the pomegranate juice, the pomegranate juice fermented with *L. plantarum*, and the bread samples was performed by freezing at −44 °C (5 °C/min) and drying for 48 h (at 5–15 mbar and −45 °C) with a FreeZone 4.5 Freeze-Drying System (Labconco, Kansas City, MO, USA). The produced freeze-dried powders were used as adjuncts in sourdough breadmaking.

### 2.4. Sourdough Breadmaking

For sourdough breadmaking, the ingredients were mixed mechanically and the doughs were molded in 1.5 L baking pans. Initially, nine sourdoughs were prepared by mixing 300 g wheat flour and 160 mL tap water with (% *w*/*w* on flour basis): (i) freeze-dried *L. plantarum* at concentrations of 1% (L1), 2% (L2), and 3% (L3); (ii) freeze-dried pomegranate juice fermented by *L. plantarum* at concentrations of 2% (L4), 4% (L5), and 6% (L6); and (iii) freeze-dried unfermented pomegranate juice 6% plus freeze-dried *L. plantarum* at concentrations of 1% (L7), 2% (L8), and 3% (L9). The amounts of adjunct used in all cases, (i), (ii), and (iii), were calculated in order to achieve similar LAB viable cell counts in the sourdoughs: 8.0 cfu/g in the L1, L3, and L7 sourdoughs, 8.8 log cfu/g in L2, L5, and L8, and 9.2 log cfu/g in L3, L6, and L9 (Table 1). All sourdoughs were left to ferment for 24 h at 30 °C.

Subsequently, nine sourdough breads were prepared containing 30% *w*/*w* (on flour basis) of the above sourdoughs. Specifically, all doughs contained 150 g of each sourdough, 500 g wheat flour, 270 mL tap water and 4 g salt. In all cases, an amount of 1% *w*/*w* (on flour basis) of pressed baker’s yeast was added. All doughs were fermented at 30 °C for 2 h, proofed at 40 °C for 60 min and baked at 230 °C for 40 min. The nine different sourdough breads contained sourdough with (% *w*/*w*, on flour basis): (i) freeze-dried *L. plantarum* (L1-L3) at concentrations of 1% (LP1), 2% (LP2), and 3% (LP3); (ii) freeze-dried fermented pomegranate juice at concentrations of 2% (POLP1), 4% (POLP2), and 6% (POLP3); and (iii) freeze-dried *L. plantarum* at concentrations of 1% (LP1PO), 2% (LP2PO), and 3% (LP3PO) with the addition of freeze-dried unfermented pomegranate juice at 6% in each case.

For comparison, bread was also made with traditional sourdough provided by a local bakery (wild microflora). The control bread contained 30% (on flour basis) of the traditional sourdough. The recipe and the procedure followed were the same as described above for the other samples. All trials were carried out in triplicate.

### 2.5. Analysis

#### 2.5.1. Microbial Cell Counts and Monitoring of Bread Spoilage

The determination of viable cell counts (colony-forming units, cfu/g) in the freeze-dried *L. plantarum* and the fermented pomegranate juice powders was carried out after homogenization of 1 g of sample in 9 mL of phosphate buffer (0.25 M solution of KH_2_PO_4_ diluted as 1.25 mL/L of distilled water) [24]. The suspensions were serially diluted, plated on MRS agar (Fluka, Buchs, Switzerland), and incubated at 37 °C for 48–72 h. In a similar manner, viable cell counts of LAB and yeasts were determined in the sourdoughs after the homogenization of 20 g of sample with 200 mL of phosphate buffer. LAB were determined as described above, while yeasts were determined on malt agar (Fluka, Buchs, Switzerland) after incubation at 30 °C for 2 days [24].

Mold and rope spoilage was monitored macroscopically on bread loaves stored in sterile plastic bags at room temperature. Mold spoilage time was determined as the day when mold growth became visible. Rope spoilage was monitored considering several parameters, such as the development of a ripe cantaloupe flavor, discoloration, and sticky threads, as described previously [25]. All determinations were carried out in triplicate.

#### 2.5.2. Organic Acids

Organic acids (lactic, acetic, formic, propionic, n-valeric and caproic) in the sourdough breads were determined using HPLC as described before [25]. Briefly, the samples (10 g) were homogenized with sterile water (90 mL) (Seward Stomacher 400 blender, London, UK) and centrifuged, and the supernatants were analyzed on a Shimadzu HPLC system (Shimadzu, Kyoto, Japan) equipped with a Shim-pack IC-A1 column, LC-10AD pump, CTO-10A oven (40 °C), and CDD-6A detector. The mobile phase (1.2 mL/min) was phthalic acid (2.5 mM) and tris(hydroxymethyl)aminomethane (2.4 mM; pH 4.0). Determination of the organic acid concentrations was carried using standard curves.

#### 2.5.3. pH and Total Titratable Acidity (TTA)

The pH values of the sourdough bread samples were measured by a Sentron Argus pH meter (Sentron Europe B.V., Roden, The Netherlands). The TTA, as mL NaOH 0.1 M consumed per 10 g of sample, was determined after homogenization of 10 g of breadcrumb with 90 mL of deionized water and titration of the supernatant with 0.1 M NaOH to a final pH of 8.5 [25].

#### 2.5.4. Specific Loaf Volume

The specific loaf volume (cm^3^/g) of the sourdough breads was measured by the rapeseed displacement method [26].

#### 2.5.5. Total Phenolic Content (TPC)

After baking, the breads were allowed to cool down to room temperature for 3 h. They were then sliced, and crumb samples were freeze-dried for 48 h (FreeZone 4.5, Labconco, Kansas City, MO, USA). Then, 1 g of freeze-dried sample was added to 20 mL of phosphate-buffered saline (PBS, pH 7.4) for 1 h under shaking at 37 °C. The extracts were separated by decanting and the residues were extracted again with 20 mL of PBS. The collected extracts were combined and stored at −20 °C until analysis for TPC and AC.

The TPC was determined by the Folin–Ciocalteu reagent method as described previously with modifications [27]. Specifically, 200 μL of sample extract was mixed with 800 μL of Folin–Ciocalteu reagent and left to react for 2 min in the dark. Then, 2 mL of sodium carbonate (7.5% *v*/*v*) was added and the volume was adjusted to 10 mL with distilled water. The mixture was left to stand for 60 min at room temperature in the dark and the absorbance was measured at 765 nm. Standard gallic acid (GA) solutions and blank were also prepared. The TPC was expressed as GA equivalents (mg GAE/100 g dried sample).

#### 2.5.6. Antioxidant Capacity (AC)

The AC of the bread extracts was evaluated by two methods: the ABTS [2,2′-azino-bis (3-ethylbenzothiazoline-6-sulfonic acid)] assay, as the Trolox Equivalent Antioxidant Capacity (TEAC), and the DPPH radical scavenging activity assay [28]. The ABTS radical cation (ABTS^•+^) stock solution was prepared by mixing 7.4 mM ABTS (in water) and 2.6 mM potassium persulfate (in water) in equal volumes. It was then incubated at room temperature for 12 h. The above solution was diluted with water to an absorbance of 0.70 ± 0.02 at 734 nm. Then, 100 μL of bread extract was mixed with 3.9 mL of the diluted ABTS^•+^ solution, and after 4 min, the absorbance was measured against a blank at 734 nm. A standard Trolox curve was also prepared, and the results were expressed as Trolox equivalents (mg TE/100 g dried sample).

Regarding the DPPH assay, in several test tubes, 3 mL of a 137.6 μM methanolic DPPH solution and various amounts of bread extract (in the range of 0.05–1 mL) were added, and the volume was fixed with methanol to 4 mL. The samples were left for 60 min in the dark and the absorbance was measured at 515 nm against aqueous methanol solution as blank. The antioxidant capacity was calculated from the Trolox calibration curve and the results were expressed as μmol TE/g of the sample (dry weight).

#### 2.5.7. Phytic Acid

Phytic acid (phytate; myo-inositol-1,2,3,4,5,6-hexakisphosphate) was measured as phosphorus released by enzymatic activity using the Megazyme test kit K-PHYT according to the manufacturer’s recommendations (Megazyme, Bray, Ireland).

#### 2.5.8. Sensory Evaluation

A blind sensory evaluation assessment was carried out for all the produced sourdough breads immediately after their production, as described previously [29]. Briefly, the sourdough breads were evaluated at a local bakery, by 20 random, untrained testers (consumer-oriented, preliminary testing), who were asked to rate their sensory properties (flavor, taste, appearance) based on a 9-point hedonic scale (1: dislike extremely, 9: like extremely). The results were recorded as average scores plus standard deviations.

#### 2.5.9. Statistical Analysis

Analysis of Variance (ANOVA) followed by Duncan’s post hoc multiple range test at the 5% level of significance was used to extract differences between the various treatments (effects of the different sourdoughs applied on the physicochemical and sensory properties of the produced breads). The analysis was carried out using the IBM SPSS Statistics 20 software.

## 3. Results

### 3.1. Viable Cell Counts in the Sourdoughs

Viable LAB and yeast cells (cfu) were enumerated in all of the prepared sourdoughs, and the results are shown in Table 1. Although both types of freeze-dried adjuncts were applied in the sourdough preparation at amounts containing viable LAB cells of approximately 7.7 log cfu/g, statistically significant differences were observed after the 24 h sourdough fermentation. Specifically, the addition of freeze-dried fermented pomegranate juice led to higher viabilities in the final sourdoughs (L4–L6) compared to the sourdoughs made with freeze-dried *L. plantarum* (L1–L3), the sourdoughs made with freeze-dried *L. plantarum* and freeze-dried, unfermented, pomegranate juice (L7–L9), and the commercial sourdough (control). The highest average viabilities were observed in sourdough L6 (11.1 log cfu/g) and L5 (10.5 log cfu/g), followed by L9 (10.1 log cfu/g), L3 (9.9 log cfu/g) and L4 (9.8 log cfu/g). On the other hand, no statistically significant differences were observed between all sourdoughs regarding the levels of yeasts, varying between 7.5 and 7.9 log cfu/g (Table 1).

### 3.2. Sourdough Bread Quality Characteristics

The specific loaf volumes (SLV) of all sourdough breads were similar (*p* < 0.05), in the average (av.) range of 2.44–2.55 cm^3^/g (Table 2). On the other hand, statistically significant differences were observed regarding the acidity of the breads. Specifically, the (av.) pH values were 4.4–4.8, and the TTA was 5.5–10.0 mL 0.01 M NaOH. The highest pH and TTA values were observed in the breads POLP3 (4.39 and 9.95, respectively), LP3PO (4.55 and 9.85, respectively), and LP3 (4.53 and 9.83 mL, respectively). The lactic acid and acetic acid concentrations were 2.1–3.0 and 0.6–1.0 g/kg, respectively, the highest being that of POLP3 (3.02 g/kg lactic acid and 0.95 g/kg acetic acid), LP3 (2.89 g/kg lactic acid and 0.92 g/kg acetic acid) and LP3PO (2.75 g/kg lactic acid and 0.89 g/kg acetic acid). Minor organic acids, such as formic, propionic, valeric, and caproic, were also found in all bread samples at lower levels (0.01–0.15 g/kg) (Table 2).

### 3.3. TPC and AC 

In Table 3, the results of the TPC and AC analysis of the sourdough breads are presented. The addition of freeze-dried pomegranate juice fermented by *L. plantarum* in the sourdough bread significantly increased (*p* < 0.05) the levels of both the TPC and AC.

Specifically, the av. TPC of POLP3 was 103 mg GAE/100 g, and the AC values were 232 mg TE/100 g (ABTS assay) and 4.7 μmol TE/g (DPPH assay), respectively, and were determined at the highest levels compared to all other sourdough breads. Accordingly, POLP2 and LP3PO exhibited higher TPC and AC levels (*p* < 0.05) compared to all other LP and LPPO sourdough breads and higher than those of POLP1 and the control bread. It seems that the freeze-dried adjuncts led to increased TPC and AC levels when they were added at concentrations higher than 3% *w*/*w* in the sourdough preparations, while the addition of the pomegranate adjunct led to the best results. It should be underlined that the addition of unfermented pomegranate juice to the sourdough breads containing *L. plantarum* (LPPO samples) increased the levels of TPC and AC compared to LP samples; however, this was not observed in comparison with the POLP samples.

### 3.4. Phytic Acid Content

**Figure 1:**
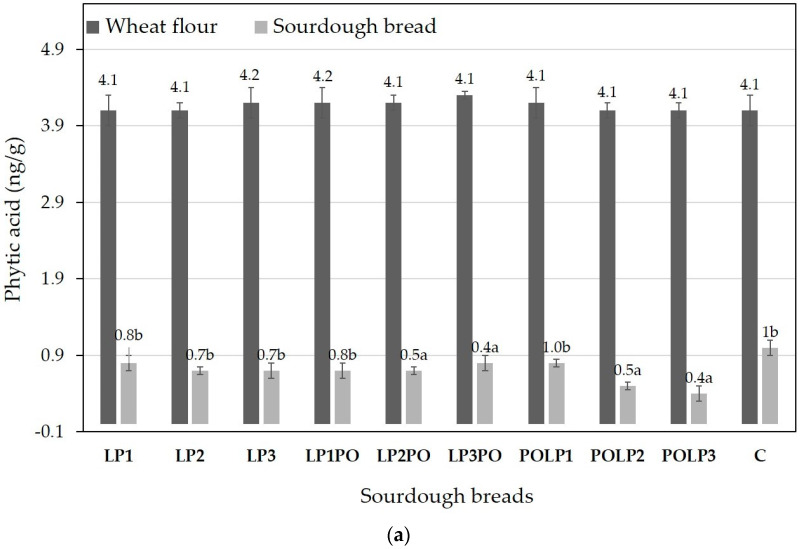

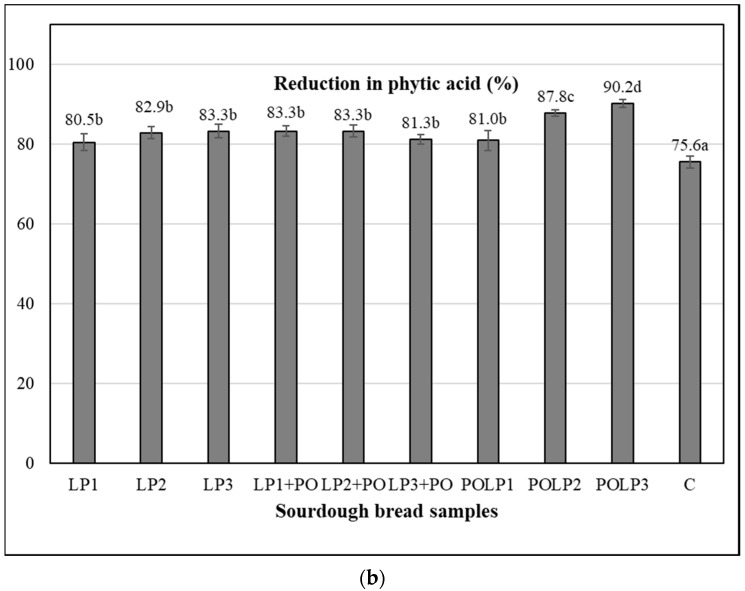
(**a**) Levels of phytic acid in the initial wheat flour and in the produced sourdough breads. (**b**) Reduction in phytic acid (%) in the produced sourdough breads. LP: sourdough breads made with different amounts of freeze-dried *L. plantarum* adjunct. LPPO: sourdough breads made with different amounts of freeze-dried *L. plantarum* plus freeze-dried, unfermented, pomegranate juice adjunct. POLP: sourdough breads made with different amounts of freeze-dried *L. plantarum* pomegranate juice adjunct. C: control bread. Different superscript letters in a bar indicate statistically significant differences (*p* < 0.05).

The content of phytic acid in the sourdough breads was low in all cases, as illustrated in Figure 1. Specifically, the sourdough breads made with the freeze-dried *L. plantarum* adjunct (LP) contained (av.) 0.7–0.8 ng/g, while those made with freeze-dried *L. plantarum* adjunct and freeze-dried, unfermented, pomegranate juice contained (av.) 0.4–0.8 ng/g and those made with freeze-dried, fermented pomegranate juice (POLP) contained 0.4–1.0 ng/g. The phytate in the initial wheat flour was 4.1–4.2 ng/g (Figure 1a). LP, LPPO and POLP breads contained less phytate (*p* < 0.05) compared to the control sourdough bread (1.0 ng/g). Higher phytate reduction (*p* < 0.05) was observed in the LP, LPPO and POLP breads compared to the control, the highest (90.2%) being that observed in the case in POLP3 bread (Figure 1b).

### 3.5. Resistance to Spoilage

**Figure 2:**
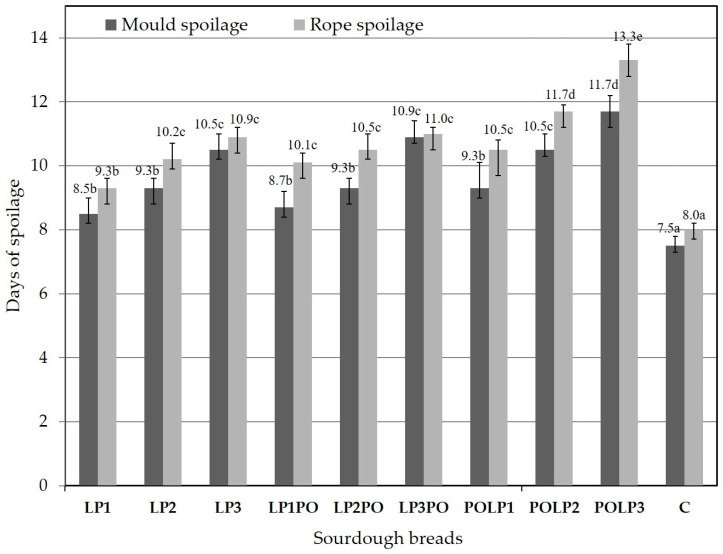
Appearance of mold and rope spoilage in the produced sourdough breads. LP: sourdough breads made with different amounts of freeze-dried *L. plantarum* adjunct. LPPO: sourdough breads made with different amounts of freeze-dried *L. plantarum* plus freeze-dried, unfermented, pomegranate juice adjunct. POLP: sourdough breads made with different amounts of the freeze-dried *L. plantarum* pomegranate juice adjunct. C: control bread. Different superscript letters in a bar indicate statistically significant differences (*p* < 0.05).

Spoilage (mold and rope) of all breads was monitored macroscopically on a daily basis, and the results are illustrated in Figure 2. All LP, LPPO and POLP sourdough breads appeared more resistant, with mold and rope spoilage appearing after the 8th and 9th day, respectively, compared to the control bread, which was spoiled after the 7th day. POLP breads were more resistant than LPPO and LP breads, while the resistance to spoilage increased with the increasing concentration of the adjunct in the sourdough. Specifically, mold and rope spoilage in POLP3 appeared after the 12th and 13th day, respectively.

### 3.6. Sensory Evaluation

The sensory properties of the sourdough breads are presented in Table 4. LP1, LP1PO and POLP1 achieved the lowest score (*p* < 0.05) compared to all other sourdough breads regarding aroma. POLP3 achieved the best score (*p* < 0.05) regarding taste. The same trend was observed when the evaluators (consumers) were asked to evaluate the overall quality of the breads. In all cases, the consumers clearly showed a preference for the sourdough breads (LP, LPPO or POLP) that contained the adjunct at the two highest concentrations (LP2-3, LPPO2-3 and POLP2-3), which were comparable (*p* < 0.05) to the control sample.

## 4. Discussion

In this study, nine different sourdough breads were prepared using sourdoughs (at 30% *w*/*w* on flour basis), which were made with different amounts of adjuncts based on a potential probiotic *L. plantarum* strain: (a) freeze-dried culture of this strain (LP breads), (b) freeze-dried culture of this strain with the addition of freeze-dried unfermented pomegranate juice (LPPO breads), and (c) freeze-dried supplement containing pomegranate juice fermented by this strain (POLP breads). In addition, a control sourdough bread sample was prepared under the same conditions, but with commercial sourdough. The quality of the new types of sourdough breads was evaluated in terms of the loaf volume, acidity, TPC, AC, phytic acid content, microbial stability (rope and mold spoilage), and sensory characteristics.

The higher LAB viable cell numbers observed in the sourdoughs made with the POLP adjunct (Table 1) may be due to the presence of components in the pomegranate juice, which positively affect the viability of *L. plantarum*. Such components may be prebiotic phenolics, such as pomegranate ellagitannins, that have been found to enhance the growth of *Enterobacteriaceae*, *Bacteroides fragilis*, clostridia, bifidobacteria, and lactobacilli, as previously reported and reviewed [30,31]. However, this was not observed to the same extent in the case of breads made with the addition of freeze-dried unfermented pomegranate juice. A possible explanation is that *L. plantarum* in the fermented pomegranate juice was adapted better. Likewise, fermented pomegranate juice possibly contained transformed antioxidant compounds exhibiting a higher antioxidant capacity compared to the unfermented juice.

Additionally, statistically significant differences were observed regarding all the examined parameters of the sourdough breads, except the specific loaf volumes (SLV) (Table 2). Therefore, the different adjuncts did not significantly affect the rising of the sourdough breads. Regarding the other parameters (pH, TTA, and organic acid concentration), it can be observed that the increasing amount of adjunct in the sourdough had a significant effect on these characteristics (Table 2). The same was observed regarding the concentrations of organic acids, with higher values found in the samples containing sourdoughs prepared with the highest amount of adjunct (LP3, LP3PO, and POLP3). Among them, POLP3 bread had a higher organic acid content. The main explanation for this result is the higher cell viability that was observed in the respective sourdough samples, leading to more effective lactic acid fermentation, as other research has proposed [23,24,25]. As discussed in a previous study [2], the levels of lactic and acetic acid are very important for the flavor of sourdough, and specific legislation requirements may be set locally for the levels of these acids (e.g., strictly pH 4.3 and acetic acid at least 900 mg/kg in France) [3]. Moreover, a quotient of fermentation (QF; ratio of lactic to acetic acid) is often determined in the sourdough bread literature [2], which is usually in the range 0.25–20 (av. 4.4) (with recommended QF < 5.0). In this study, the average QF range for all treatments was in the range 2.8–3.6.

The production of organic acids by various LAB species and their effect on the sensory properties and shelf life as well as their levels in sourdough breads have recently been reviewed [3]. LAB species including *L. paracasei* K5 [3,25] and *Pediococcus pentosaceus* SP2 [24], isolated by our research group from dairy products, were also recently used to produce sourdough breads under similar conditions. These starters were used as fresh cultures, and in freeze-dried and immobilized forms (on prebiotic cereal matrices). It was observed that these strains produced similar or slightly higher amounts (maximum av. values) of acetic acid (1.0–1.1 g/kg) and lactic acid (~2.9–3.2 g/kg).

Regarding the TPC and AC of the sourdough breads, it was observed that the addition of both adjuncts led to improvements in these nutritional features; the best results were obtained in the case of the fermented pomegranate juice adjunct. This can be explained by the fact that pomegranate juice is rich in flavonoids and other antioxidant phenolic compounds. Moreover, the antioxidant composition of pomegranate juice was previously found to be ameliorated by lactic acid fermentation [23]. Similar findings were reported in other recent studies, where supplements with high phenolic and antioxidant contents were applied in breadmaking, leading to increases in the TPC and AC [9,32,33,34], while the use of powdered fruit and vegetables in breadmaking is to be applied commercially. It should be underlined that the composition of fermented pomegranate juice with *L. plantarum* in compounds that exhibit high AC was at higher levels, and this was reflected in the produced sourdough breads. However, unfermented pomegranate juice contains antioxidant compounds, the transformation of which to other forms of potentially higher AC is not likely to take place to the same extent as in the fermented pomegranate juice by *L. plantarum*. This has been explained by other researchers who have claimed that lactic acid fermentation of pomegranate juice enhances the AC of the final product compared to the unfermented juice [23].

Another reason for the ameliorated AC and TPC in the sourdough breads produced in this study may be the specific strain applied. Specifically, *L. plantarum* strains exhibit high versatility and flexibility due to their multifunctional enzyme activities. They are considered to be very effective regarding their biotransformation ability of bound phenols to soluble compounds with higher AC and bioavailability [35,36,37]. Specifically, in [37], a 48% increased TPC content (from 209 to 310 mg GAE/100 g) was observed in sourdough inoculated with *L. plantarum* after 72 h of fermentation, which was attributed to the transfer of water-soluble polyphenols during fermentation, from the flour to the sourdough, with the aid of the LAB proteolytic activity. According to [38], the TPC of sourdough, after 24 h of fermentation with *L. plantarum*, was increased by 83% (from 42 to 77 mg GAE/100 g). In this study, the TPC was increased by 49% and 87% from the lowest to the highest adjunct addition, for LP and POLP breads, respectively (Table 3).

Regarding the AC of sourdoughs fermented by *L. plantarum,* in [38], it was found to be equal to 62 mg TE/100 g after 24 h of fermentation (3.4 times higher than that of unfermented sourdough), and in [37], 3.6 mmol TE/100 g was determined after 72 h, corresponding to a 83% increase. It is therefore clear that *L. plantarum* strains can increase the AC of sourdoughs, affecting the levels of bioactive components through metabolic activities that release antioxidant compounds.

The use of pomegranate juice fermented by the strain *L. plantarum* ATCC 14917 as a freeze-dried adjunct in sourdough breadmaking has not been previously reported. A comparison can be made with studies involving the use of pomegranate seed powder (PSP) as an additive in the flour that will be used for the preparation of bread [21,39]. According to [21], the TPC of samples containing PSP ranged from 402 to 466 mg GAE/kg, depending on the fermentation time, while AC was found to be in the range 1059–2575 μmol TE/kg, compared to 280–376 mg GAE/kg and 525–1017 μmol/kg, for the control samples, respectively. Both the TPC and AC of the breads made with PSP increased with the increasing percentage of PSP in the flour [39].

Finally, it should be noted that the assay based on the Folin–Ciocalteu reagent (mixture of the heteropoly phosphomolybdate and phosphotungstate acids) is not specific for phenolic compounds, since other compounds such as ascorbic acid and other vitamins, thiols (such as cysteine and glutathione), nucleotide bases, etc., are active in the assay. Therefore, the TPC assay based on the Folin–Ciocalteu reagent provides a measurement of the AC of a sample rather than a real estimation of total phenolics [40] and is suitable and commonly used for comparing samples or treatments.

Phytic acid is considered an antinutrient in bread because it makes complexes with various mineral ions, leading to decreased bioavailability and deficiencies in the human diet [41,42]. There is evidence that sourdough fermentation plays a significant role in reducing the phytate content in bread, while LAB, including *L. plantarum* strains, are known to exhibit extracellular phytase activity [42]. Likewise, an increase in the acidity of bread has been correlated with a decrease in phytate [43]. Indeed, a higher reduction in the phytate content in this study was observed with an increasing concentration of adjunct in the sourdoughs (Figure 1). Specifically, POLP3, which had the lowest phytate content, had the highest acidity in terms of TTA as well as lactic and acetic acid content (Table 2).

The better resistance to rope and mold spoilage of the POLP breads compared to LPPO and LP breads and the increased resistance with an increasing concentration of sourdough adjunct can also be attributed to the activity of LAB and the higher levels of acidity (Table 2). The antimicrobial role of organic acids and other LAB metabolites is well established [44]; however, more research is needed to identify certain antimicrobial compounds produced by specific LAB, such as *L. plantarum* strains. Pomegranate constituents, such as phenolics, may also exhibit antimicrobial action [2,36]. The enhanced viability of *L. plantarum* observed in the POLP sourdoughs, as already discussed, may also explain their better resistance to rope and mold spoilage.

Finally, given that the consumers clearly showed a preference for breads LP2-3, LPPO2-3, and POLP2-3 (Table 4), the proposed nutritious adjuncts can be used in sourdough breadmaking, leading to products with acceptable sensory properties, comparable to similar commercial breads, when added in the initial sourdough at amounts of around 3% *w*/*w* on the flour basis.

## 5. Conclusions

The results of the present study showed that adjuncts based on the strain *L. plantarum* ATCC 14917 can be successfully applied in sourdough breadmaking, leading to products of improved nutritional quality (in terms of TPC, AC, and phytate reduction), and with good sensory properties. The adjunct that contained freeze-dried pomegranate juice fermented by *L. plantarum* gave the best results overall, while all breads presented higher resistance against mold and rope spoilage compared to the control due to their higher acidity and organic acid contents and possibly due to the presence of antimicrobial pomegranate phenolics or other *L. plantarum* metabolites. In addition, the application of the proposed adjuncts in freeze-dried, powdered form facilitates their commercial application, e.g., as formulations available in sachets for the bakery sector.

## Figures and Tables

**Table 1:** Yeast and LAB counts in the produced sourdoughs.

Sourdough	Log cfu/g	
	LAB	Yeasts
Freeze-dried *L. plantarum*		
L1	8.9 ± 0.2 ^a2^	7.5 ± 0.3 ^a1^
L2	9.4 ± 0.1 ^b2^	7.7 ± 0.2 ^a1^
L3	9.9 ± 0.2 ^c2^	7.5 ± 0.1 ^a1^
Freeze-dried pomegranate juice fermented by *L. plantarum*		
L4	9.8 ± 0.1 ^c1^	7.9 ± 0.3 ^a1^
L5	10.5 ± 0.1 ^d1^	7.9 ± 0.1 ^a1^
L6	11.1 ± 0.1 ^e1^	7.5 ± 0.2 ^a1^
Freeze-dried *L. plantarum* and freeze-dried pomegranate juice		
L7	8.9 ± 0.1 ^a2^	7.6 ± 0.3 ^a1^
L8	9.3 ± 0.1 ^b2^	7.7 ± 0.2 ^a1^
L9	10.1 ± 0.2 ^c2^	7.6 ± 0.1 ^a1^
Control	8.7 ± 0.2 ^a^	7.7 ± 0.1 ^a1^

LAB: lactic acid bacteria. cfu: colony-forming units. Different superscript letters in a column and different numbers between (L1, L4, L7), (L2, L5, L8) and (L3, L6, L9) indicate statistically significant differences (*p* < 0.05).

**Table 2:** Physicochemical parameters of the produced sourdough breads.

Sourdough Bread	pH	TTA (mL NaOH 0.1 M)	SLV (cm^3^/g)	Organic Acids (g/kg Bread)					
				Lactic	Acetic	Formic	Propionic	*n*-Valeric	Caproic
Freeze-dried *L. plantarum* (LP; 1–3%)									
LP1 (1%)	4.79 ± 0.02 ^d^	5.85 ± 0.05 ^b^	2.45 ± 0.05 ^a^	2.13 ± 0.05 ^a^	0.59 ± 0.02 ^b^	0.07 ± 0.01 ^a^	0.03 ± 0.01 ^a^	0.04 ± 0.01 ^a^	0.03 ± 0.01 ^a^
LP2 (2%)	4.66 ± 0.03 ^c^	7.22 ± 0.07 ^c^	2.52 ± 0.05 ^a^	2.54 ± 0.07 ^b^	0.78 ± 0.03 ^c^	0.08 ± 0.01 ^a^	0.05 ± 0.01 ^a^	0.05 ± 0.01 ^a^	0.03 ± 0.01 ^a^
LP3 (3%)	4.53 ± 0.02 ^b^	9.83 ± 0.05 ^d^	2.51 ± 0.07 ^a^	2.89 ± 0.07 ^c^	0.92 ± 0.02 ^d^	0.11 ± 0.01 ^b^	0.10 ± 0.01 ^b^	0.07 ± 0.01 ^a^	0.03 ± 0.01 ^a^
Freeze-dried *L. plantarum* (LP; 1–3%) and freeze-dried pomegranate juice (PO; 6%)									
LP1PO	4.78 ± 0.03 ^d^	5.82 ± 0.05 ^b^	2.42 ± 0.05 ^a^	2.15 ± 0.04 ^a^	0.54 ± 0.02 ^b^	0.06 ± 0.02 ^a^	0.03 ± 0.01 ^a^	0.04 ± 0.01 ^a^	0.03 ± 0.01 ^a^
LP2PO	4.68 ± 0.02 ^c^	7.19 ± 0.05 ^c^	2.50 ± 0.04 ^a^	2.51 ± 0.05 ^b^	0.76 ± 0.02 ^c^	0.09 ± 0.01 ^a^	0.04 ± 0.01 ^a^	0.05 ± 0.01 ^a^	0.03 ± 0.01 ^a^
LP3PO	4.55 ± 0.02 ^b^	9.85 ± 0.05 ^d^	2.50 ± 0.05 ^a^	2.75 ± 0.05 ^c^	0.89 ± 0.04 ^d^	0.12 ± 0.01 ^b^	0.09 ± 0.02 ^b^	0.08 ± 0.03 ^a^	0.03 ± 0.01 ^a^
Freeze-dried pomegranate juice fermented by *L. plantarum* (POLP; 2–6%)									
POLP1 (2%)	4.83 ± 0.03 ^d^	5.53 ± 0.07 ^a^	2.44 ± 0.07 ^a^	2.10 ± 0.05 ^a^	0.55 ± 0.02 ^b^	0.06 ± 0.01 ^a^	0.004 ± 0.01 ^a^	0.03 ± 0.01 ^b^	0.03 ± 0.01 ^a^
POLP2 (4%)	4.56 ± 0.02 ^b^	7.93 ± 0.05 ^e^	2.53 ± 0.05 ^a^	2.62 ± 0.07 ^b^	0.81 ± 0.02 ^c^	0.11 ± 0.01 ^b^	0.011 ± 0.01 ^b^	0.10 ± 0.01 ^b^	0.03 ± 0.01 ^a^
POLP3 (6%)	4.39 ± 0.02 ^a^	9.95 ± 0.05 ^f^	2.50 ± 0.07 ^a^	3.02 ± 0.05 ^d^	0.95 ± 0.03 ^d^	0.15 ± 0.01 ^c^	0.012 ± 0.01 ^b^	0.12 ± 0.01 ^b^	0.07 ± 0.01 ^b^
Control	4.75 ± 0.03 ^c^	6.10 ± 0.08 ^c^	2.55 ± 0.04 ^a^	2.18 ± 0.05 ^a^	0.77 ± 0.02 ^a^	0.07 ± 0.01 ^a^	0.03 ± 0.01 ^a^	0.04 ± 0.01 ^a^	0.03 ± 0.01 ^a^

TTA: Total titratable acidity. SLV: Specific loaf volume. Different superscript letters in a column indicate statistically significant differences (*p* < 0.05).

**Table 3:** Total phenolic content (TPC) and antioxidant activity (AC) (on a dry-weight basis) of the produced sourdough breads.

Sourdough Bread	TPC (mg GAE/100 g)	AC	
		ABTS (mg TE/100 g)	DPPH (μmol TE/g)
Freeze-dried *L. plantarum* (LP; 1–3%)			
LP1 (1%)	49.0 ± 8.2 ^a1^	174.7± 7.3 ^a1^	3.0± 0.2 ^a2^
LP2 (2%)	63.8± 3.5 ^b2^	184.8± 5.5 ^a3^	3.2± 0.1 ^a3^
LP3 (3%)	73.4± 4.5 ^c3^	199.6± 6.4 ^b2^	3.7± 0.2 ^a3^
Freeze-dried *L. plantarum* (LP; 1–3%) and freeze-dried pomegranate juice (PO; 6%)			
LP1PO	54.4 ± 1.7 ^a1^	180.1± 3.9 ^a1^	3.2± 0.1 ^a2^
LP2PO	78.3± 4.1 ^c1^	195.8± 3.1 ^b2^	3.7± 0.1 ^a2^
LP3PO	90.1± 4.6 ^d2^	202.4± 4.9 ^b2^	4.1± 0.1 ^b2^
Freeze-dried pomegranate juice fermented by *L. plantarum* (POLP; 2–6%)			
POLP1 (2%)	54.7± 4.1 ^a1^	185.4± 5.3 ^a1^	3.5± 0.1 ^a1^
POLP2 (4%)	85.8± 3.5 ^d1^	214.9± 5.1 ^c1^	4.4± 0.1 ^c1^
POLP3 (6%)	102.4± 4.3 ^e1^	231.5± 4.2 ^d1^	4.7± 0.1 ^d1^
Control	52.8± 5.1 ^a^	182.7± 6.1 ^a^	3.2± 0.1 ^a^

Different superscript letters in a column and different numbers between (LP1, LP1PO, POLP1), (LP2, LP2PO, POLP2) and (LP3, LP1PO, POLP3) indicate statistically significant differences (*p* < 0.05).

**Table 4:** Sensory evaluation of the produced sourdough breads.

Sourdough Bread	Aroma	Taste	Appearance	Overall Quality
Freeze-dried *L. plantarum* (LP; 1–3%)				
LP1 (1%)	8.4 ± 0.1 ^a^	8.4 ± 0.1 ^a^	8.5 ± 0.1 ^a^	8.5 ± 0.1 ^a^
LP2 (2%)	8.8 ± 0.1 ^b^	8.8 ± 0.1 ^b^	8.9 ± 0.1 ^b^	8.8 ± 0.1 ^b^
LP3 (3%)	8.8 ± 0.1 ^b^	8.9 ± 0.1 ^b^	8.8 ± 0.1 ^b^	8.8 ± 0.1 ^b^
Freeze-dried *L. plantarum* (LP; 1–3%) and freeze-dried pomegranate juice (PO; 6%)				
LP1PO	8.5 ± 0.1 ^a^	8.5 ± 0.1 ^a^	8.5 ± 0.1 ^a^	8.5 ± 0.1 ^a^
LP2PO	8.8 ± 0.1 ^b^	8.7 ± 0.1 ^b^	8.8 ± 0.1 ^b^	8.8 ± 0.1 ^b^
LP3PO	8.8 ± 0.1 ^b^	8.9 ± 0.1 ^b^	8.9 ± 0.1 ^b^	8.8 ± 0.1 ^b^
Freeze-dried pomegranate juice fermented by *L. plantarum* (POLP; 2–6%)				
POLP1 (2%)	8.3 ± 0.1 ^a^	8.6 ± 0.1 ^a^	8.5 ± 0.1 ^a^	8.5 ± 0.1 ^a^
POLP2 (4%)	8.7 ± 0.1 ^b^	8.9 ± 0.1 ^b^	8.9 ± 0.1 ^b^	8.8 ± 0.1 ^b^
POLP3 (6%)	9.1 ± 0.1 ^b^	9.2 ± 0.1 ^c^	8.9 ± 0.1 ^b^	9.0 ± 0.1 ^b^
Control	8.9 ± 0.1 ^b^	8.9 ± 0.1 ^b^	8.9 ± 0.1 ^b^	8.9 ± 0.1 ^b^

Different superscript letters in a column indicate statistically significant differences (ANOVA, Duncan’s multiple range test, *p* < 0.05).

## Data Availability

Not applicable.
